# Chagas Disease: Still Many Unsolved Issues

**DOI:** 10.1155/2014/912965

**Published:** 2014-06-29

**Authors:** José M. Álvarez, Raissa Fonseca, Henrique Borges da Silva, Cláudio R. F. Marinho, Karina R. Bortoluci, Luiz R. Sardinha, Sabrina Epiphanio, Maria Regina D'Império Lima

**Affiliations:** ^1^Department of Immunology, Biomedical Sciences Institute, University of São Paulo, 05508-000 São Paulo, SP, Brazil; ^2^Department of Parasitology, Biomedical Sciences Institute, University of São Paulo, 05508-000 São Paulo, SP, Brazil; ^3^Department of Biological Sciences, UNIFESP (Campus Diadema), 09972-270 Diadema, SP, Brazil; ^4^Hospital Israelita Albert Einstein, 05652-000 São Paulo, SP, Brazil; ^5^Department of Clinical and Toxicological Analyses, Faculty of Pharmaceutical Sciences, University of São Paulo, 05508-000 São Paulo, SP, Brazil

## Abstract

Over the past 20 years, the immune effector mechanisms involved in the control of *Trypanosoma cruzi*, as well as the receptors participating in parasite recognition by cells of the innate immune system, have been largely described. However, the main questions on the physiopathology of Chagas disease remain unanswered: “Why does the host immune system fail to provide sterile immunity?” and “Why do only a proportion of infected individuals develop chronic pathology?” In this review, we describe the mechanisms proposed to explain the inability of the immune system to eradicate the parasite and the elements that allow the development of chronic heart disease. Moreover, we discuss the possibility that the inability of infected cardiomyocytes to sense intracellular *T. cruzi* contributes to parasite persistence in the heart and the development of chronic pathology.

## 1. A Brief Overview of Chagas Disease

Chagas disease is caused by* Trypanosoma cruzi* and represents an important health problem in Latin America, with approximately 8 million chronically infected people [1]. Recently, as a consequence of human migrations, Chagas disease has become a potential public health issue in developed countries, and significant increases in confirmed cases have been reported in the USA, Canada, Europe, Japan, and Australia [2]. The invasion of the human host frequently occurs by the passage through damaged skin or intact mucosa of metacyclic trypomastigotes released with the feces of infected triatomines after their blood meal. Alternatively, infection through other routes, such as oral, congenital, and blood transfusion/organ transplantation, also occurs. Because it is an obligate intracellular parasite,* T. cruzi* can be found in the vertebrate host as amastigotes, the intracellular replicative form, and as extracellular trypomastigotes circulating freely in the blood and tissues. The infection has a self-limiting acute phase, with patent (or subpatent) parasitemia, which goes unnoticed in many infected individuals. At this stage, the parasites actively replicate in many different cell types, such as macrophages; smooth, striated, and cardiac muscle cells; adipocytes; and cells of the central nervous system [3].

While a small proportion of patients succumbs to the acute phase of the disease, the development of the adaptive immune response typically provides control of the* T. cruzi* infection, albeit nonsterile control. Failing to completely eradicate the parasite, individuals remain infected for life and establish a dynamic equilibrium with the parasite that results in different clinical outcomes. Thus, while many chronically infected individuals remain in the asymptomatic indeterminate phase, a significant proportion (30–35%) of patients develop the cardiac or digestive manifestations of chronic disease: cardiomyopathy that may lead to congestive heart failure, arrhythmia and, eventually, patient death, and esophageal or colonic megasyndromes. These are irreversible pathological changes that occur despite parasite scarcity. Recapitulating human chagasic myocarditis, mice surviving long-term infection by certain stocks of* T. cruzi* develop chronic myocardial lesions [4, 5].

## 2. The Host Immune Response against* T. cruzi*

The immune system is well equipped to detect and control* T. cruzi* parasites through the combined effect of diverse branches of the immune response. CD4^+^ and CD8^+^ T cells, as well as B cells, contribute to control the parasite through cytokine secretion, cellular cytotoxicity, and specific antibody production [6–8]. From the end of the acute phase and throughout chronic infection,* T. cruzi*-specific IgG antibodies actively participate in the removal of extracellular parasites released from ruptured tissue nests, an effect that presumably occurs by promoting parasite phagocytosis by macrophages and neutrophils. In addition, specific IgG antibodies mediate the removal of blood trypomastigotes, a clearance process in which complement and mononuclear phagocytes from the liver, spleen, and lungs appear to be involved [9–11]. The IFN-*γ* produced by activated CD4^+^ and CD8^+^ T cells, NK cells, and CD4^−^CD8^−^ *γ* *δ* T cells plays a crucial role in parasite elimination [12, 13]. IFN-*γ* potentiates the effector activity of macrophages by inducing the transcription of the inducible nitric oxide synthase (iNOS) gene, notably increasing the production of nitric oxide, which has a potent effect on* T. cruzi* killing [14, 15]. In addition, IFN-*γ* promotes the immunoglobulin switch to IgG subclasses with high opsonizing and complement-activating potential. Lastly, cytotoxic CD8^+^ T cells also contribute to* T. cruzi* control through the recognition and destruction of cells that harbor intracellular forms of the parasite [16].

## 3. Limitations to the Innate and Acquired Immune Responses That Contribute to Parasite Persistence

One of the most intriguing questions of human and experimental* T. cruzi* infection is why the immune system fails to totally eradicate the parasite. At first glance, the inability of the infected host to attain sterility suggests that the immune effector activity directed against the parasite is insufficient or inappropriate due to defective activation of the specific immune response or excessive regulation of this response.

In this context, we outline in this section the different escape mechanisms employed by* T. cruzi* parasites and discuss the hypothesis generated to explain an immune system failure. At the beginning of the infection (before development of the parasite-specific response),* T. cruzi* trypomastigotes escape lysis by the complement system, an evasion strategy that results from the presence of complement-regulatory molecules on the parasite surface [17]. In addition, internalized parasites of diverse* T. cruzi* strains escape the phagocytic vacuole of unprimed resident macrophages [18], a strategy that relies on a variety of molecules with antioxidant properties [19, 20]. Nevertheless, as infection progresses, these two evasion strategies are largely circumvented by the development of the specific humoral response and the induction of macrophage activation by IFN-*γ* and other cytokines. Because* T. cruzi* strains display different levels of antioxidant activity that directly correlate with strain virulence [21], it remains unclear whether IFN-*γ* confers effective macrophage protection against any* T. cruzi* parasite or results in different degrees of intracellular parasite destruction for different isolates.

Pattern recognition receptors (PRRs), such as toll-like receptors (TLRs) 2, 4, 7, and 9, nucleotide-binding oligomerization domain-like receptor (NOD) 1 and NATCH, LRR, and PyD domains-containing protein 3 (NLRP3) have been shown to participate in* T. cruzi* detection by macrophages and dendritic cells [22–27]. However, a deficient innate immune response due to poor PRR signaling by pathogen-associated molecular patterns (PAMPs) has been proposed as a mechanism involved in parasite escape. This hypothesis is supported by data showing that, compared with mice infected by wild-type* T. cruzi* parasites, mice infected with transgenic* T. cruzi* expressing* Salmonella typhimurium* flagellin (fliC) display an increased innate response mediated by macrophages and dendritic cells and notably enhanced adaptive immunity [28]. More importantly, in the chronic phase, mice infected with fliC-transgenic* T. cruzi* display notably reduced parasite levels in relation to those infected with wild-type parasites.

Parasite persistence has also been attributed to the relatively slow development of* T. cruzi*-specific CD8^+^ T effector cells [29], a phenomenon explained by diverse factors, including the postulated poor PAMP activity of* T. cruzi* [28] and the induction of a strong Fas expression on* T. cruzi*-specific CD8^+^ T cells [30]. Another possible reason for the failure to acquire sterile immunity is the clonal dominance of the lymphocyte response to* T. cruzi* infection. This is illustrated by the observation that the CD8^+^ T cell response to the abundantly expressed* T. cruzi* antigen amastigote surface protein-2 (ASP2) is restricted to a small number of clones [31]. Narrowing the scope of parasite peptides that are recognized by CD8^+^ T cells may impair complete parasite eradication during chronic infection and the control of reinfection, which frequently occurs in endemic areas.

**Figure 1:**
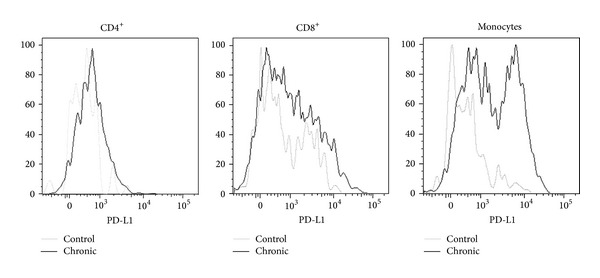
Expression of PD-L1 by infiltrating leukocytes in a chronically infected heart. C3H/HePAS mice were infected with 4 × 10^5^ Sylvio X10/4 trypomastigotes obtained from LLCMK2 cultures. At day 400 postinfection, mice were sacrificed, and the heart tissue was digested with collagenase to isolate the infiltrating leukocytes. PD-L1 expression was analyzed by flow cytometry. Heart leukocytes from a pool of age-matched noninfected mice were also included.

An alternative mechanism that restricts the efficiency of adaptive immunity in eliminating* T. cruzi* parasites is the negative regulation of effector lymphocytes because of persistent stimulation. The senescence of CD4^**+**^ T cells [32] and exhaustion of CD8^**+**^ T cells that infiltrate the heart or striated muscle of chronically infected hosts have been observed during chronic infection [33, 34]. In this context, it was suggested that the parasite can survive inside myocardial or striated muscle cells because following migration to the tissues CD8^+^ T cells lose their cytotoxic and IFN-*γ*-producing capacities [35]. Also suggesting the negative regulation of the effector response, Albareda and cols [36] reported that CD4^**+**^ T cells from patients with long-term chronic infection are primarily monofunctional, whereas in children in the early chronic stage of infection, multifunctional responses are also observed. Last, the expression of PD-1 and PD-L1 regulatory molecules has been shown to downmodulate the effector activity of CD4^**+**^ and CD8^**+**^ T cells in* T. cruzi*-infected mice [37]. While PD-L1-PD-1 regulatory interaction was observed in the acute phase of infection, its roles in limiting parasite elimination and permitting the perpetuation of lesions in the chronic phase remain to be determined. In this respect, our data showing the intense expression of PD-L1 in the heart-infiltrating leukocytes of mice chronically infected by Sylvio X10/4* T. cruzi* parasites suggest the involvement of this regulatory circuit in parasite persistence (Figure 1).

The mechanisms of parasite escape discussed above refer to limitations of the innate or acquired protective immune response to* T. cruzi* that appear to operate in all infected individuals. However, in addition to these general elements, we must consider that individuals differ in the intensity/effectiveness of their anti-*T. cruzi* humoral and cellular responses, a consequence of polymorphisms in genes associated with the immune response [38]; these polymorphisms influence the intensity of the anti-*T. cruzi* effector activity, yielding different levels of residual parasites and undesired tissue lesions in chronically infected human patients. In addition, these polymorphisms influence the residual parasite distribution in different tissues/organs, an element closely connected to the development of the different forms of the disease.

The isogenic strains of mice also differ in the immune response to* T. cruzi*. These differences are often critical for acute phase survival [39] and most likely determine the parasite load in the chronic phase. However, not a single mouse strain has been reported to promote the complete elimination of the parasite. Therefore, regardless of the mouse/parasite strain combination, the inevitable outcome of murine infection by* T. cruzi* appears to be parasite persistence, a result analogous to that observed in human patients.

## 4. Only a Proportion of* T. cruzi*-Infected Individuals Show Chronic Pathologies

A significant proportion of chronically infected individuals develop the cardiac and digestive forms of the disease, but the largest fraction present the indeterminate form. Importantly, although many indeterminate patients remain asymptomatic for the rest of their lives, it is estimated that, each year, 2.5% of infected individuals evolve from the indeterminate to the clinical forms [40]. Chronic chagasic cardiomyopathy (CCC) represents the main cause of death in* T. cruzi*-infected patients. Moreover, this clinical form represents an important social burden in terms of lost labor hours and hospital costs. The situation of CCC patients is worrisome because the specific anti-*T. cruzi* drugs, currently limited to benznidazol and nifurtimox, show limited efficacy in chronically infected patients.

Because of parasite scarcity in the inflamed heart, CCC was long considered an autoimmune disease directed against self-epitopes showing cross-reactivity with parasite antigens [41]. According to this view, lesions were thought to occur as a result of T cell reactivity against myosin and other heart-derived proteins [42] as well as humoral reactivity to the beta-1-adrenergic and M2 cholinergic receptors, leading to autonomic nervous system imbalance [43]. However, in the last 20 years, cumulative evidence has promoted a change in our understanding of this process. First, immunohistochemistry data showed that, in patients with CCC, the level of* T. cruzi* antigen correlated with the intensity of the inflammatory infiltrate [44]. Furthermore,* T. cruzi* DNA was found in the heart of diseased CCC patients but not in the heart of patients with the indeterminate form [45]. In contrast, patients with megaesophagus, one of the digestive forms of the disease, displayed positive PCR for* T. cruzi* kinetoplast DNA in the esophagus [46]. Confirming the human studies, in mice chronically infected by* T. cruzi*, we previously observed that live parasites were only detected in the heart of mice with cardiomyopathy, although these mice displayed subpatent blood parasite levels similar to those in mice with no heart pathology [47]. Based on the data from these and other important reports, at present, it is largely agreed that the inflammatory infiltrate in CCC is caused by a response directed toward locally persisting parasites [48]. Still, because Chagas disease is a highly heterogeneous process influenced by host genetics, we cannot discard that the immune response in the heart of CCC patients could include, besides leukocyte reactivity towards locally persisting parasites, different degrees of autoreactivity. Furthermore, it is possible that a proportion of CCC patients shows cardiac pathology in the absence of locally persisting infection. This last possibility is illustrated by a recent report using bioluminescence imaging, in which mice chronically infected with luciferase expressing* T. cruzi* parasites display mild heart inflammation in the absence of local parasitism [49]. Further studies on the contribution of autoimmunity are required for a full comprehension of CCC physiopathology. These studies may eventually reveal alternative therapeutic approaches to attenuate heart tissue inflammation in chagasic patients.

Nonetheless, because most data suggest the association of chronic cardiac pathology with local parasite persistence, CCC is presently understood as an expression of the host's incapacity to totally eliminate the heart parasitism, a particularity of the host's failure to eradicate* T. cruzi* from the organism. This leads us to question why heart parasitism occurs in only a fraction of* T. cruzi* chronically infected individuals.

## 5. Where Does* T. cruzi* Persist in the Chronically Infected Host?

The tissue distribution of* T. cruzi* parasites varies among chronically infected individuals. As indicated above, patients with cardiomyopathy and megaesophagus harbor parasites in the heart and esophagus, respectively. However, are there in these patients, as well as in those with the indeterminate form, other locations where the parasite is relatively safe from complete elimination by the immune system?* A priori*, because parasite persistence is the rule, it is reasonable to hypothesize that immune-privileged* T. cruzi* reservoirs must exist in all chronically infected hosts. One of these parasite niches could be the central adrenal vein, which, in autopsy studies of patients with chronic Chagas disease, has been found to frequently harbor amastigote nests; further, the presence of these nests shows a close correlation with heart pathology parameters such as the intensity of leukocyte infiltration and myocardial fibrosis [50]. Additionally, both brown and white adipose tissues, as well as the colon and stomach, have been described as locations where* T. cruzi* parasites chronically persist [49, 51, 52]. Moreover, because of parasite persistence in the cardiac tissue of CCC patients, the heart could be one of these niches for a fraction of the infected population.

Another issue is whether parasite dissemination through the blood or by spreading from neighboring tissues occurs in the chronic host. Are the* T. cruzi* found in the hearts of CCC patients the consequence of chronic phase dispersion from other tissues or do they result from the perpetuation of heart colonization during the acute phase? This is an important question because chronic phase reinvasion could readdress the problem of heart parasite persistence outside the cardiac tissue.

**Figure 2:**
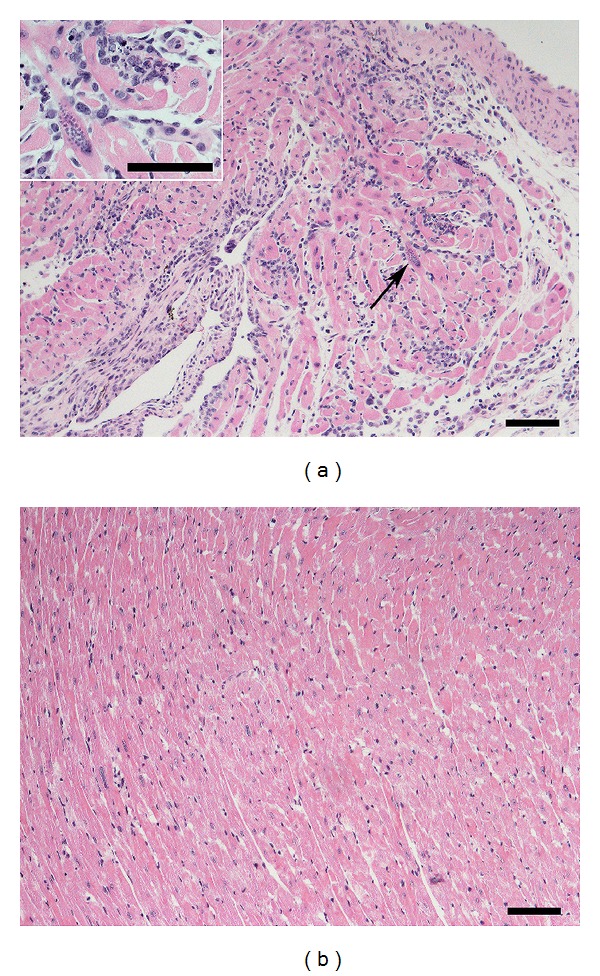
Parasite persistence is not the necessary outcome of heart infection by* T. cruzi* parasites. C57BL/6 mice were infected with 10^3^ *T. cruzi* blood trypomastigotes of the Y strain. At days 18 (a) and 180 (b) postinfection, mice were sacrificed, and the heart tissue was formalin-fixed, included in paraffin, and stained with hematoxylin/eosin. Arrow in the figure shows an amastigote nest, which is magnified at the insert. Bars in figures correspond to 200 *μ*m and to 100 *μ*m at the insert.

On the other hand, it is important to note the observation made by different research groups that mice from certain strains can eliminate* T. cruzi* from the heart. This occurs in C57BL/6 mice infected with a sublethal dose of Y strain parasites; these mice exhibit strong leukocyte infiltration with the presence of amastigote nests in the heart in the late acute phase but no signs of infection or pathology in this organ in the chronic phase (Figure 2). This observation indicates that parasite persistence is not the necessary outcome of heart infection. Moreover, these results open the possibility that the indeterminate group of human patients might include patients in whom the cardiac infection was resolved in addition to individuals in whom the heart was never colonized by the parasite.

## 6. Elements Involved in Parasite Persistence in the Heart or Other Tissues: Parasite Tropism

*T. cruzi* displays a broad heterogeneity, being currently classified in six different groups (I-VI) [53] that show discrete correlations with the sylvatic or peridomestic forms of transmission as well as with the occurrence (or lack thereof) of different chronic pathologies. Mixed infections by multiple* T. cruzi* isolates are frequently observed in chronic chagasic patients.

Parasite tropism was originally defined as the preferential invasion of a cell type by a* T. cruzi* clone/isolate. Nevertheless, because the infected host exhibits considerable variability in its tissue responses to the parasite, tropism has to be redefined as the outcome of the interaction of a defined* T. cruzi* clone/isolate in a particular individual, a process largely dependent on the parasite and host genetics. The importance of the host in the development of chronic heart pathology by a single* T. cruzi* parasite is clearly illustrated in the murine model of infection by parasites of the* T. cruzi* clone Sylvio X10/4, which results in chronic cardiomyopathy in C3H/HePAS mice but not in C57BL/6 or A/J mice [47].

Therefore, while (at least for mice and men) the lack of a spontaneous cure and, consequently, the persistence of the parasite are general problems associated with* T. cruzi* infection, independent of parasite and host diversity, the development of chronic heart disease appears to be limited to particular host-parasite combinations.

## 7. Is Parasite Persistence Merely the Result of a Deficit in the Local Immune Response?

Different research groups have analyzed the heart-infiltrating leukocytes of CCC patients and mice with chronic cardiomyopathy [54–58]. These studies have yielded valuable data regarding the distribution of leukocyte populations, surface marker expression, and the production of cytokines, chemokines, and other mediators. Nonetheless, the gathered information did not help to determine whether a local immune deficit exists because the characteristics of an effective local immune response are undefined. This is because in those chronic settings in which an effective response could eventually occur, such as in chronically infected mice with no cardiac pathology or in patients with the indeterminate form, there are by definition no heart infiltrates to dissect. Therefore, if there is a defect in the local immune response in the heart of CCC patients, it has yet to be found.

Immune response analysis of the blood of patients with the cardiac and indeterminate forms has been used as an indirect means of searching for the presumed local immune defect, aiming to reveal a special immune signature that would explain why the parasite persists in the heart of CCC patients. Remarkably, these studies have revealed that the production of IFN-*γ* and other proinflammatory cytokines is higher in CCC patients than in indeterminate form patients [59–61] and that asymptomatic patients exhibit augmented T_REG_ numbers and IL-10 levels [59, 62]. Because a proinflammatory response is considered the appropriate approach to eliminate* T. cruzi* parasites, these results conflict with the hypothesis that parasite evasion is the result of a deficient immune response. Furthermore, while systemic studies do not suggest a local deficit in the anti-*T. cruzi* immune response of CCC patients, they do indicate that these patients display an aggressive deregulated response that may explain the presence of tissue damage in the affected heart [63].

**Figure 3:**
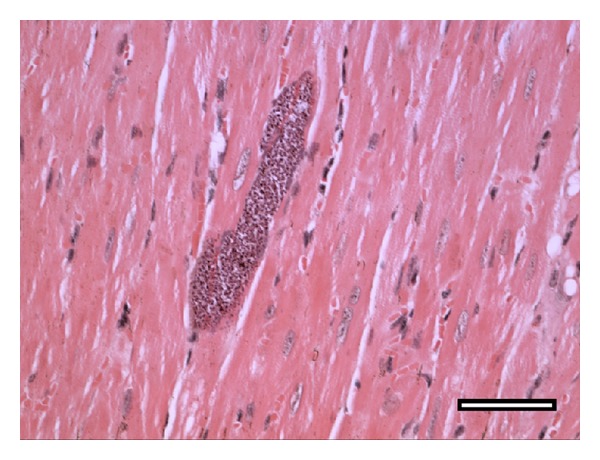
Lack of sensing of amastigote nests could contribute to parasite perpetuation and pathology in the chronically infected heart. Lack of sensing could occur independently of the origin of cardiomyocyte-invading trypomastigotes, that is, from a local ruptured nest or metastasis from a distant niche. The heart picture shown corresponds to a C3H/HePAS mouse infected for more than 200 days with 10^6^ Sylvio X10/4 trypomastigotes. The tissue section was stained with hematoxylin-eosin. Bar corresponds to 100 *μ*m (reproduced from C. R. F. Marinho,* Microbes and Infection* [64] with permission from Elsevier).

What, then, is missing? How can we explain why patients with greater proinflammatory responses to* T. cruzi* are those with lower heart parasite control? An interesting possibility is that* T. cruzi* persistence in the heart (and in other relatively immunoprivileged locations) is not merely the consequence of a defective local immune response but that it, to a large extent, derives from the parasite's ability to remain unnoticed inside the structural cells, safe from the effector activity of cytotoxic CD8^+^ T cells [64]. Illustrative evidence of this possibility is shown in Figure 3, where an amastigote nest can be observed amid myocardial fibers in a mouse infected for more than 200 days, that is, in an animal with a high level of anti-*T. cruzi* immune effector activity. While a notable feature of this picture is the nest size, the most impressive aspect is the absence of leukocytes surrounding it. This suggests a deficiency in signaling for leukocyte recruitment, a condition that allows the parasite to temporarily evade the immune system. Anecdotally, the presence of heart amastigote nests undetected by the inflammatory response was described by Vianna [65] in 1911, one year after the discovery of Chagas disease. From these observations, we can postulate that, independently of the cardiomyocyte-invading parasites originating from a local ruptured nest or a distant niche, the failure to attain sterile immunity in the heart may to a large extent result from an intrinsic or acquired deficit of the cardiomyocytes in sensing the intracellular parasite. Because leukocyte recruitment requires infected cardiomyocytes to signal the presence of an infection through the secretion of chemotactic molecules, we can speculate that, in patients with chronic cardiac disease, this mechanism is largely suppressed. Meanwhile,* T. cruzi*-infected neonatal cardiomyocytes have been shown* in vitro* to transcribe the genes for the chemokines MCP-1, RANTES, KC/GRO, MIP-2, MIG, and IP-10 together with those for the cytokines TNF-*α* and IL-1*β* [66]. A way to reconcile these* in vitro* and* in vivo* findings is that the failure of cardiomyocyte signaling* in vivo* could be an adaptive trait that develops during the course of infection in only certain parasite-host combinations. The possibility that* T. cruzi *interferes with the physiology of infected structural cells is supported by other studies. In this manner, cruzipain, an enzyme abundantly found in* T. cruzi* parasites, has been shown to interfere with cardiomyocyte apoptosis through activation of the NF*κ*B and PI3K/Akt and MEK1/ERK1/2 pathways in the host cell, which lead to increased expression of antiapoptotic Bcl-2 molecules and increased arginase expression [67]. It is therefore conceivable that the crosstalk of the intracellular parasite with the signaling pathways of these structural cells might negatively impact the production of chemotactic molecules.

A deficit in intracellular parasite sensing, although important, is insufficient to guarantee* T. cruzi* evasion in the chronically infected heart. This is the case given that, sooner or later, an undetected amastigote nest will spontaneously disrupt, releasing extracellular parasites that, after detection by antibodies, will cause complement and resident macrophages to generate mediators for leukocyte recruitment. Thus, for long-term parasite perpetuation to occur, it is predictable that a small fraction of nest-released trypomastigotes will reinvade relatively distant cardiomyocytes, where they may remain unnoticed and out of the reach of cytotoxic CD8^+^ T cells in the newly formed infiltrate.

As stated above, defective parasite sensing by cardiomyocytes could be an adaptive process of the heart tissue that develops with the length of infection. This process most likely exhibits a great degree of variability, reflecting the genetics of the host and the parasite. Moreover, its occurrence in the cardiac tissue is not surprising because the heart is a vital organ that must have special mechanisms designed to protect its integrity.

## 8. Local Parasite Destruction versus Immunopathology

Theoretically, if two hosts are unable to control tissue parasites, the one with a greater local inflammatory response will pay a higher price by provoking greater damage of the infected tissue [68]. Therefore, if defective local sensing occurs in CCC patients, strong local immune responses would clearly represent a detrimental factor in the induction of pathology, thus explaining the reported associations between high levels of cardiac dysfunction and genotypes associated with high reactivity [69, 70]. Furthermore, the inverse correlation observed in CCC patients between the intensity of electrocardiogram abnormalities and IL-10 plasma levels [59] reinforces this view. That is, to respond strongly when there is a gap in local* T. cruzi* control clearly represents a deleterious manner of dealing with the parasite.

Entering into a persistent cycle of an intense local effector response with no resolution is, to a certain extent, a form of autoaggression, considering the high price paid by the organism in terms of tissue damage. It is not, however, an aggression specifically directed against self-antigens but the unwanted price for unceasingly attempting to completely eliminate a small number of parasites that persist in a fraction of chronically infected individuals. Paradoxically, to protect heart integrity, cardiomyocytes may allow parasite persistence, which indirectly results in tissue damage every time a nest breaks open and new leukocyte infiltrates are formed.

A deficit in the interaction of* T. cruzi* parasites with tissue structural cells could also be involved in parasite persistence at locations other than the heart. This could occur in any infected patient, independent of whether the infection is cardiac, digestive, or indeterminate. In contrast to the heart, however, in many of these locations, the bystander tissue damage resulting from the persistent immune reaction against parasites might not be sufficient to compromise the function of the infected tissue.

## Future Perspectives

While extensive research has deciphered the local and systemic immune responses of chronically* T. cruzi*-infected hosts in the last two decades, future studies will need to focus on the* in vivo* interaction of parasites with structural cells, in both the heart and other tissues. Although these studies currently face great technical challenges, they will be of great importance to improve our knowledge about Chagas disease pathology.
